# Acute Hepatitis C After Penile Stem Cell Injection

**DOI:** 10.7759/cureus.31858

**Published:** 2022-11-24

**Authors:** Sahil Zaveri, Ariana Tagliaferri, Bhavin Shah, Gabriel Melki, Patrick Michael

**Affiliations:** 1 Internal Medicine, State University of New York Downstate Health Sciences University, New York, USA; 2 Internal Medicine, St. Joseph's University Medical Center, Paterson, USA; 3 Family Medicine, Mercy Health St. Rita's, Lima, USA; 4 Medicine, St. Joseph's University Medical Center, Paterson, USA

**Keywords:** immunocompetent, liver injury, stem cell transplant, penile injection, hepatitis c (hcv) infection

## Abstract

Hepatitis C is a viral infection that is transmitted via blood or other bodily fluids and usually manifests as a chronic infection. We present a unique case of acute hepatitis C from a penile stem cell injection. Although previous cases have reported the reactivation of chronic hepatitis C after hematopoietic stem cell transplantation, it is uncommon for hepatitis C to present acutely, especially in an immunocompetent patient. To our knowledge, this is the first case of acute hepatitis C after a penile stem cell injection.

## Introduction

Hepatitis C causes chronic infection by inducing an inflammatory response in the liver, resulting in accumulated damage over time [1,2]. The hepatitis C virus (HCV) is an enveloped, positive-sense, single-stranded ribonucleic acid (RNA) virus of the Flaviviridae family [1,2]. It is most commonly transmitted from contact with blood and plasma in the setting of shared needles or blood transfusions [1,2]. Many cases of acute hepatitis C are likely underreported and underdiagnosed due to the clinical quiescence of the disease in its acute state [3]. Patients that present within a 6-month window from the time of exposure are considered acute cases, as opposed to chronic cases, which present after 6 months [3]. It can be difficult to distinguish an acute from a chronic hepatitis C infection because the abnormalities seen in laboratory studies are typically the same [4]. In general, labs that can be used to diagnose hepatitis C are serum HCV RNA, anti-HCV IgG, detection of anti-HCV IgM, measurement of the anti-HCV IgG avidity index, elevated liver enzymes and/or changes to the viral load [3,4]. Serum antibodies can take up to 12 weeks to form after transmission [4]. Thus, the HCV RNA is the most accurate way to detect acute hepatitis, especially if tested early after a known exposure [4]. This was observed previously in patients with acute hepatitis C who had lower HCV RNA levels than those with known and chronic HCV after seroconversion in the initial 10 weeks following exposure [5].

Approximately 52% of untreated symptomatic patients will clear their viral infection spontaneously; however, many will progress to a chronic state if not treated [3]. Treatment options include ribavirin, interferon, interferon-free regimens, NS3/4A inhibitors, and NS5B inhibitors [6]. The mechanisms of action for these drugs inhibit viral assembly and viral RNA polymerase and/or directly block viral replication [6]. Treatments are targeted at specific genotypes and viral load, which should be tested at the time of diagnosis [6]. Herein, we present a patient diagnosed with acute hepatitis C after a penile stem cell injection for erectile dysfunction.

## Case presentation

**Figure 1:**
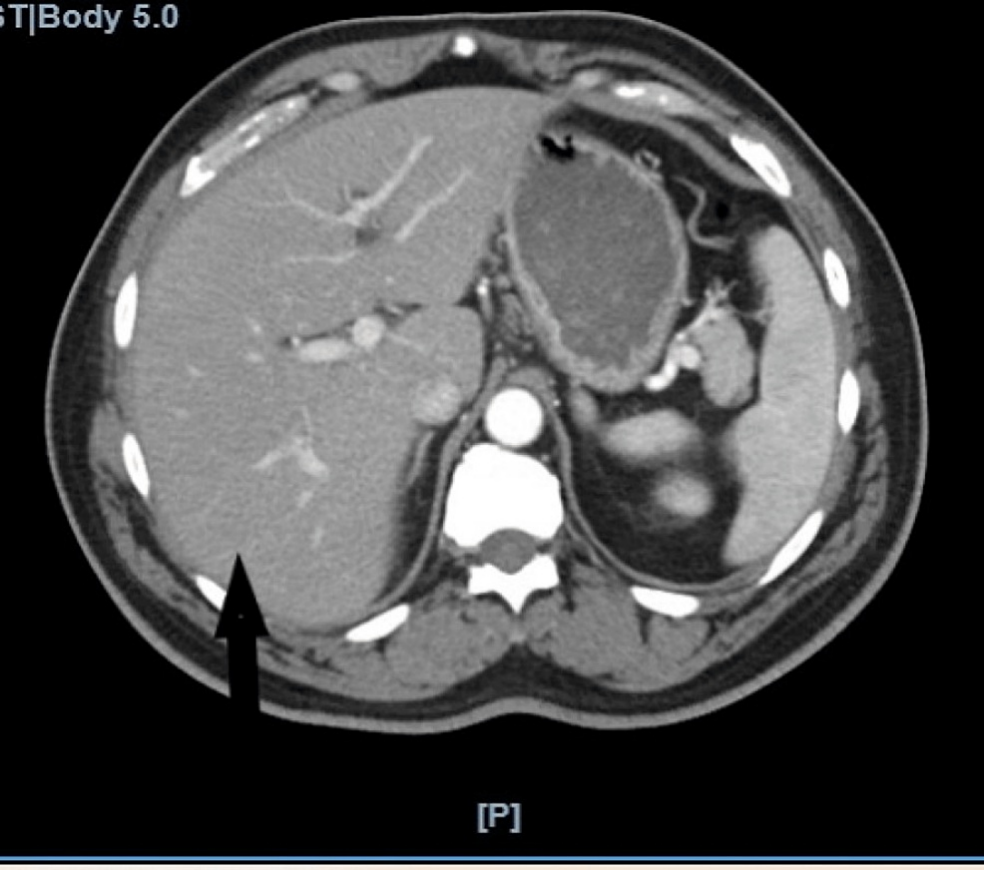
Computerized tomography of the abdomen and pelvis with intravenous contrast. Axial view showing numerous non-specific and small hypodensities visualized in the right hepatic lobes (black arrow).

A 58-year-old male with a past medical history of erectile dysfunction was referred to the emergency department (ED) by his primary care physician for scleral icterus. The patient traveled to the Dominican Republic 3 weeks prior to the presentation. While in the Dominican Republic, he underwent a penile stem cell injection for erectile dysfunction and ate food from street vendors. This subsequently caused an episode of nausea, nonbilious emesis, and watery diarrhea lasting three days. Although these symptoms subsided, he began to have chills and general malaise lasting 14 days before the patient presented to his doctor for evaluation. On arrival to the ED, he had a temperature of 36.6ºC, heart rate of 74 beats per minute, blood pressure of 183/104 mm/Hg, respiratory rate of 16, and was saturating 99% on room air. On physical examination, he had jaundice, diffuse abdominal tenderness without rigidity or guarding, mild ascites, and hepatomegaly. Through further history, the patient reported exclusive unprotected sexual activity with his wife, consumption of 2-3 beers every weekend, and no history of blood transfusions or intravenous drug use. The review of systems was otherwise negative. His labs were remarkable for mild leukocytosis (8.6 K/μL), abnormal liver enzymes in a mixed hepatocellular and cholestatic pattern (alanine transaminase (ALT) 1046 U/L, aspartate transaminase (AST) 570 U/L, alkaline phosphatase (ALP) 163 U/L), conjugated hyperbilirubinemia (total bilirubin 20.8 mg/dL, direct bilirubin 14.3 mg/dL), and elevated prothrombin time (16.3 seconds). He was admitted to the general medical floors for further workup of suspected hepatitis and was initially treated with N-acetylcysteine infusion until further testing resulted. Polymerase chain reaction (PCR) testing was negative for Epstein-Barr virus (EBV), cytomegalovirus (CMV), and herpes simplex virus 1/2 (HSV). His autoimmune markers were negative for actin smooth muscle antibody, antinuclear antibodies (ANA), liver-kidney microsomal antibody, and M2 mitochondrial antibody. His toxicology screen was entirely negative. However, a hepatitis panel revealed a history of hepatitis A (positive total antibodies), a positive anti-HCV antibody of 11 S/Co, and HCV RNA qualitative of 8050 IU/mL. Hepatitis B core IgM, core total, and surface antibody qualitative results were all negative and non-reactive. The patient underwent a portal vein ultrasound which showed normal patent veins with normal flow and waveform patterns. Computed tomography (CT) of the abdomen and pelvis with intravenous contrast revealed only non-specific and small hypodensities in the liver lobes (Figure 1). He was ultimately diagnosed with acute hepatitis C based on the final HCV RNA, genotype 1a. The patient was managed with supportive care and was medically optimized for discharge on amlodipine 10 mg oral daily and lisinopril 10 mg oral daily due to persistent hypertension. On scheduled follow-up in the gastroenterology clinic 2 weeks later, his jaundice had resolved, bilirubin levels had normalized, and the patient was entirely asymptomatic. Hepatitis and autoimmune panel results are summarized in Table 1.

**Table 1:** Viral and autoimmune panel laboratory results. PCR: polymerase chain reaction; DNA: deoxyribonucleic acid

Serum markers	Results
Epstein–Barr Virus PCR	Not Detected
Cytomegalovirus PCR	Not Detected
Herpes Simplex Virus 1 DNA, PCR	Not Detected
Herpes Simplex Virus 2 DNA, PCR	Not Detected
Actin Smooth Muscle Antibody	17 units (Ref. Range 0-19 units)
Anti-Nuclear Antibody	Negative
Anti-Mitochondrial Antibody	< 20 units (Ref. Range 0-20 units)
Hepatitis A IgM Antibody	Negative
Hepatitis A Total Antibody	Positive
Hepatitis B Core IgM Antibody	Negative
Hepatitis B Core Total Antibody	Negative
Hepatitis B Surface Antibody Qualitative	Non-reactive
Hepatitis B Surface Antigen	Negative
Hepatitis C Genotype	1a
Hepatitis C Quantity	8050 IU/mL
Hepatitis C Virus Antibody	>11.0 s/co (Ref. range 0.0-0.9 s/co)

## Discussion

Reactivation of hepatitis C is a well-known consequence of bone marrow and hematopoietic transplantation in immunocompromised individuals [1,2]. Previous studies have identified a propensity for immunocompromised individuals to acquire acute hepatitis C infections and to develop hepatitis C reactivation. [7]. In one study, those with hematological malignancies undergoing chemotherapy and transplant recipients had an 11% higher chance of HCV reactivation than those with solid tumor neoplasms [7]. Other studies have demonstrated a relationship between HCV reactivation and hematological malignancies, such as acute myeloid leukemia, chronic myeloid leukemia, non-Hodgkin’s lymphoma, and multiple myeloma [8]. Despite an acquisition rate of 29% for acute hepatitis C and a reactivation rate of approximately 12% in this demographic, there were no significant differences in mortality due to hepatic morbidities [9].

Although it is known that acute hepatitis C and reactivated hepatitis C are seen in immunocompromised patients and/or from transplant recipients, this is the first presentation of an acute hepatitis C episode in an immunocompetent patient who underwent a penile stem cell injection [10]. A potential vector of transmission is from contaminated needles in the Dominican Republic, as all other risk factors were negative [9,10]. No information was able to be obtained about the particular stem cell donor as this patient had completed this procedure in the Dominican Republic. In a former study, the genotype was 1a, which is also prevalent among immunocompetent patients [10]. Interestingly, this genotype was associated with higher carcinogenic properties in immunocompetent patients [10,11]. Genotypes 2 and 3 were more prominent in immunocompromised patients, as the study revealed they were in greater occurrence in hepatocellular carcinoma and lymphoma as they had three times the risk of cancer than genotype 1 [12]. Our patient’s case is also unique because he was symptomatic in the initial days following transmission. This is rare for patients to develop symptoms in the acute phase [11,12].

Chronic hepatitis can lead to liver cirrhosis, acute liver decompensation, and hepatocellular carcinoma [4]. Early treatment and detection of hepatitis C, even in the acute phase, can prevent chronic and long-term sequelae [5]. Apart from small hypodensities, the CT and ultrasound were not indicative of chronic liver disease. Although one may have chronic hepatitis C in the absence of cirrhosis, the lack of laboratory or imaging stigmata of chronic liver disease suggests that this is in fact acute hepatitis C [5]. Current literature shows that the viral load may be lower in acute seroconversions compared to the viral load seen in chronic infections [5]. Our patient’s viral load was 8050 IU/mL at the time of diagnosis, which further suggests that this is an acute hepatitis C infection [13].

## Conclusions

While there are studies connecting sexual practices, intravenous drug use, and blood transfusions as vectors for transmission of hepatic viral illnesses, rarely is the connection made between immunocompetent individuals with penile stem cell injections and the onset of acute hepatitis C. Although it can be challenging to differentiate between acute and chronic hepatitis C, as patients may not be symptomatic and laboratory studies may be ambiguous, clinicians should consider lower HCV RNA as a more sensitive and specific means of diagnosing acute hepatitis C. To prevent long-term complications, early diagnosis and intervention are imperative.
